# Hyperintensionality for Logics

**DOI:** 10.1007/s10670-025-01047-y

**Published:** 2025-12-13

**Authors:** Maria Beatrice Buonaguidi

**Affiliations:** Department of Philosophy, King’s College London, Strand, London, WC2R 2LS UK

## Abstract

A logic is said to be hyperintensional when it admits connectives or operators creating hyperintensional contexts, i.e. sentential contexts not respecting the intersubstitutivity *salva veritate* of co-intensionals. Odintsov and Wansing (J Philos Logic 50(1):33–56, 2021) suggest a formal criterion for classifying a logic as hyperintensional based on whether its consequence relation is self-extensional. I argue that satisfaction of Odintsov and Wansing’s criterion is neither necessary nor sufficient for characterising a logic as hyperintensional. Indeed, the criterion classifies as hyperintensional logics which are not meant to capture hyperintensional behaviour, and fails to classify as hyperintensional logics which admit some forms of hyperintensional behaviour. I suggest several possible criteria for a logic to count as hyperintensional, arguing that each of them corresponds to a different understanding of hyperintensionality for logics. Then, I weigh these criteria against a family of logics, which display different “nuances” of hyperintensional behaviour. Finally, I gesture towards a diagnosis of hyperintensionality for logics.

## Introduction

On most accounts, hyperintensionality is characterized as a feature of sentential contexts.^1^ A context or position in a sentence is defined to be hyperintensional if and only if it does not license the unrestricted intersubstitutivity *salva veritate* of sentences sharing the same intension. Here and in the following, we take “intension” to usually mean a function from sentences to possible worlds. Essentially, hyperintensional contexts are contexts which are fine-grained enough to be sensitive to changes in the semantic content of a sentence, even when these are not reflected by a change in intension.

Standard examples of hyperintensional sentential contexts are given by verbs such as “seeing”, or “believing” (Barwise & Perry, 1983, p. 175). Indeed, the following sentences share the same intension, i.e. are true at the same set of possible worlds:

- The sky is blue in London.
- The sky is blue in London and it is raining or it is not raining in New York.

However, the sentences “I see that the sky is blue in London” and “I see that the sky is blue in London and it is raining or it is not raining in New York” may differ in truth value.

As Duži and Jespersen note (2015, p. 527) defining hyperintensionality in terms of the failure of intersubstitutivity of co-intensionals “does not address the question of just ‘how hyper’ hyperintensions are”. Indeed, the definition does not specify what *can* be substituted salva veritate inside a hyperintensional context, and what understanding of “co-intensionality” is taken to be enough to license hyperintensional behaviour.

Again, according to Duži and Jespersen, the problem of finding a positive characterization of hyperintensionality is at least twofold:

“One task becomes how to decide which of all the various contexts are the hyperintensional ones. This is in effect the problem of determining which contexts exceed the resources of modal logic. Another task becomes how to decide which hyperintensions can be substituted for which other hyperintensions inside which hyperintensional contexts. This is in effect the problem of providing a positive definition of the calibration of hyperintensional individuation. Just how close the discrimination ought to be remains an entirely open and lively research question. Obviously, one theory may want to adopt more than one measure of hyperintensional individuation to suit different theoretical purposes” (Duži & Jespersen, 2015, p. 526).

The latter task may be seen to be dependent on the former. By identifying, relative to some theoretical purpose, one or more precise criteria which pick out contexts exceeding the resources of modal logic, we can determine which hyperintensions are intersubstitutable salva veritate. In other words, by pinpointing what exactly counts as a hyperintension in some precise setting, we can single out the correct level of fine-grainedness for a given context.

Then, the question of “how hyper hyperintensions are” will depend largely on what level of fine-grainedness beyond the resources of modal logic we take hyperintensional contexts to capture. As Leitgeb (2019) notes, we may identify a sort of “continuum” between intension and interpreted syntax, where hyperintensional contexts should identify, if any, one or more natural stopping points.

$$ \text {Extension } \hookrightarrow \text { Intension } \hookrightarrow \underbrace{\dots }_\text {natural stopping point?} \hookrightarrow \text { (Interpreted) syntax} $$

The present paper will advance towards answering the question of what the measure of hyperintensional individuation, or the “stopping point in the continuum” should be, when considering logics as our theoretical purpose. A logic can be said to be hyperintensional whenever it admits connectives or operators which create hyperintensional contexts. The question of what logics create hyperintensional contexts has been somewhat debated in recent and less recent literature: Leitgeb describes his logic ${\textbf {HYPE}}$ as “a background system for modal operators that create hyperintensional contexts” (Leitgeb, 2019), while Sedlar introduces a logic ${\textbf {H}}$ of all hyperintensional models (Sedlár, 2019).^2^

Depending on how the notion of intension is understood, logical contexts which do not respect the intersubstitutivity salva veritate of co-intensionals can be described in a few possible ways, such as:

- Contexts which do not respect the intersubstitutivity salva veritate of *logical equivalents* (Cresswell, 1975; Jago, 2014);
- Contexts which do not respect the intersubstitutivity salva veritate of *classical logical equivalents* (Leitgeb, 2019);
- Contexts which do not respect the intersubstitutivity salva veritate of *necessary equivalents* (Nolan, 2014).

It is safe to assume that both Cresswell and Nolan, who formulate, respectively, the first and the third criterion, are talking about hyperintensional contexts generated by operators added to the background of classical logic, such as a “seeing” or “believing”. In particular, Nolan seems to consider intensions just to be the contexts spanned by a necessity modality supplementing classical logic. On the other hand, Leitgeb’s remark is clearly to be interpreted in a non-classical context, since it applies the notion of hyperintensionality to the logic ${\textbf {HYPE}}$.

While all these possible specifications of what counts as an intension—and hence what counts as a context in which co-intensionals fail to be intersubstitutable—may seem equivalent, this is not the case. As Williamson notes (Williamson, 2006), when we consider languages in which necessitation does not preserve logical truth, the failure of intersubstitutivity salva veritate of logical equivalents does not correspond to the failure of intersubstitutivity salva veritate of necessary equivalents. When we consider non-classical settings, this phenomenon is even more evident. Let’s consider some intuitive examples .

- I believe that *3 + 3 = 6*;
- I believe that *3 + 3 = 6* and the continuum hypothesis is either true or not true.

The propositions “*3+3=6*” and “*3+3= 6* and the continuum hypothesis is either true or not true” are both classically logically equivalent and necessarily equivalent (if we consider the truth of mathematical statements to be necessary). However, the two propositions are clearly not intuitionistically equivalent.

When working in classical logic, then, the sentential context generated by “I believe that” is hyperintensional in the sense that it does not license the intersubstitutivity salva veritate of logical equivalents (and, obviously, of classical logical equivalents) nor that of necessary equivalents. On the other hand, the same context in intuitionistic logic is not hyperintensional with respect to (intuitionistic) logical equivalents, but it is hyperintensional with respect to classical logical equivalents.

Then, when trying to carve out a measure of hyperintensionality applicable to non-classical logics or axiomatic theories, the informal definitions of hyperintensional contexts that have been suggested in the literature need to be precisified. The aim of this paper will be to identify the differences between several ways in which we can specify failure of intersubstitutivity of co-intensionals and, accordingly, to formulate several formal criteria of hyperintensionality.

I will argue that we cannot identify only one correct formal criterion corresponding to one of the intended specifications of intension for failure of intersubstitutivity, given their divergence in some non-classical settings. On the contrary, there can be different coexistent and incomparable degrees of hyperintensionality: the determination of which contexts are hyperintensional depends on what exactly makes them “hyper”, rather than how hyper they are.

Much has been written on the so-called granularity problems in hyperintensionality: there is little consensus on how fine-grained or coarse-grained hyperintensional contexts should be. As argued by Bjerring and Schwarz (2017), different phenomena and different contexts seem to call for different levels of granularity. It has been discussed how to order these, with Hornischer (2017) suggesting that granularity levels should be arranged according to a partial ordering. While it is certain that there are some incomparable notions of hyperintensionality and incomparable ways to understand hyperintensional contexts, I will argue that these are not only incomparable in the standard sense of a partial order, but also qualitatively different, in the sense that different criteria capture properly different ways to understand hyperintensionality.

The paper will be structured as follows: in Sect. 2, I will introduce and analyse a hyperintensionality criterion for logical consequence relations introduced by Odintsov and Wansing (2021). This criterion is meant to correspond to the identification of hyperintensional contexts as contexts which do not license the intersubstitutivity of logical equivalents salva veritate. I will argue that Odintsov and Wansing’s characterisation is not satisfactory, since fulfilment of their criterion is neither necessary nor sufficient for a logic to be hyperintensional.

I then consider a further criterion for hyperintensionality which can be extrapolated by a definition of hyperintensional context due to Timothy Williamson, and formulated over the classical modal logic ${\textbf {S5}}$. This criterion will be meant to capture the failure of intersubstitutivity salva veritate of necessary equivalents. Similarly to Odintsov and Wansing’s criterion, this Williamson-style criterion overgenerates in some cases and undergenerates in others. I consider cases in which the two criteria diverge, showing that they are incomparable.

In Sect. 3, I will introduce two novel criteria of hyperintensionality. The former will be meant to capture the notion of hyperintensionality as failure of intersubstitutivity salva veritate of classical logical equivalents. The latter will provide a weaker criterion of hyperintensionality, adapting the idea that, when considering non-classical logics, intensions should be defined with respect to the logico-semantical background under consideration, i.e. with respect to a given non-classical semantics.

In Sect. 4, as a case study, I will weigh these four criteria against a family of logics which display different nuances of hyperintensional behaviour. The case study will highlight a correlation between different “flavours” of hyperintensional behaviour and different degrees of failure of contraposition.

## Setting the Landscape

I first recall some basic logical notions and some assumptions which will be used throughout the article, and clarify the language and notation I will adopt. In the interest of simplicity, I will limit the following discussion to propositional logics. However, the criteria for hyperintensionality I will outline, and the arguments supporting and comparing different criteria can be easily extended to first-order settings.

### Notational Preliminaries

Let $\mathcal {L}$ denote a propositional language with a set of logical symbols $\{\wedge , \vee , \lnot , \rightarrow \}$ and a set $\texttt {Prop}$ of propositional variables, whose elements will be denoted using lowercase Latin letters *p, q, r… *. Let $\texttt {Form}$ be the set of formulae of $\mathcal {L}$, defined inductively in a standard fashion. Let $\mathcal {L}_\bot$ be the language resulting from adding the propositional constant *⊥ * to $\mathcal {L}$. When necessary, I will disambiguate between $\texttt {Form}_{\mathcal {L}}$ and $\texttt {Form}_{\mathcal {L}_{\bot }}$, for formulae of $\mathcal {L}$ and $\mathcal {L}_\bot$, respectively.

I will use lowercase Greek letters $\varphi , \psi , \chi \dots$ as metavariables for formulae of $\mathcal {L}$ and $\mathcal {L}_\bot$, specifying the language where appropriate. The symbol $\varphi \leftrightarrow \psi$ is defined as usual as $(\varphi \rightarrow \psi ) \wedge (\psi \rightarrow \varphi )$. The material conditional $\varphi \supset \psi$ is defined as $\lnot \varphi \vee \psi$. Material equivalence $\varphi \equiv \psi$ is defined as $(\varphi \supset \psi ) \wedge (\psi \supset \varphi )$. We indicate by $\sigma [\varphi /p]$ the result of uniformly replacing the propositional variable *p* by the formula *φ * in *σ *.

A logic $L \subseteq \texttt {Form}$ is a set of formulae, called axioms, closed under modus ponens and a substitution rule.

$$ \text {MP}\frac{\quad \varphi \rightarrow \psi \qquad \varphi }{\psi } \qquad \text {SR}\frac{\quad \vdash \varphi (p)}{\quad \vdash \varphi (b/p)}\quad \text {with } b \in \mathcal {L} $$

In the following, however, I will adopt the convention of omitting the substitution rule and formulating logics using axiom schemata. Let $\Gamma \subseteq \texttt {Form}$ and $\varphi \in \texttt {Form}$. Then, the consequence relation for *L* can be characterised as a binary relation $\vdash _{{\textbf {L}}} :\wp (\texttt {Form}(\mathcal {L})) \longrightarrow \texttt {Form}(\mathcal {L})$ such that $\Gamma \vdash _{{\textbf {L}}} \varphi$ iff *φ * can be derived from $\Gamma \cup L$ by a finite number of applications of the rules of inference for *L*. We write $\varphi \dashv \vdash _{{\textbf {L}}} \psi$ to indicate that *φ * and *ψ * are interderivable in *L*.

Logics can also be characterised in terms of their consequence relation. In the following, I will make extensive use of this characterisation. The consequence relation $\vdash _{{\textbf {L}}}$ is said to be Tarskian iff, for any formula $\varphi \in \texttt {Form}$ and set of formulae $\Gamma \subseteq \texttt {Form}$, it is *reflexive* (if $\varphi \in \Gamma$ then $\Gamma \vdash \varphi$), *monotonic* (if $\Gamma \vdash \varphi$ and $\Gamma \subseteq \Gamma '$ then $\Gamma '\vdash \varphi$) and *transitive* (if $\Gamma \vdash \psi$ and $\Gamma , \psi \vdash \varphi$, then $\Gamma \vdash \varphi$) (Tarski, 1936).

#### Definition 2.1

A propositional logic is characterised as a pair $\textbf{L} = \langle \mathcal {L}, \vdash _{\textbf{L}}\rangle$, where $\mathcal {L}$ is a propositional language, and $\vdash _{\textbf{L}}$ is a Tarskian consequence relation for $\mathcal {L}$ such that if $\Gamma \vdash _{{\textbf {L}}} \varphi$ then $\sigma (\Gamma ) \vdash _{{\textbf {L}}} \sigma (\varphi )$ for any substitution *σ * in $\mathcal {L}$.^3^

I am making a few simplifying assumptions which restrict the sample of logics I will be considering. Hence, the analysis which will be provided in what follows will have to be considered as a partial one, and as a starting point for more comprehensive characterisations of hyperintensionality.

First of all, I will restrict my discussion to logics with a primitive conditional connective. This is because some of the criteria which I will present are only applicable to logics with a conditional. The minimal requirement which I consider for a conditional connective to act as such is that it satisfy modus ponens.

Similarly, I am taking a logic to be a set of sentences closed under uniform substitution and modus ponens, and I am restricting my discussion to logics with a Tarskian consequence relation.

This means that the criteria I will outline in the following will not be straightforwardly applicable to some substructural logics such as the logic ${\textbf {ST}}$ (Cobreros et al., 2012), which display a non-transitive consequence relation, as well as logics which can only be presented via their inferences, such as Strong Kleene logic ${\textbf {SK}}$. While it is outside the scope of this paper to generalise the present discussion to logics with non-Tarskian consequence relations and logics with no theorems, the question of how to correctly define hyperintensionality for these logics is left to future work.

A notion which will be crucial in what follows is that of self-extensionality, or congruentiality, for a consequence relation (see Wójcicki, 1979; Humberstone, 2011).

#### Definition 2.2

(Self-ext) A logic $\textbf{L} = \langle \mathcal {L}, \vdash _{\textbf{L}}\rangle$ is **self-extensional** iff, whenever $\varphi \dashv \vdash _{\textbf{L}} \psi$, then $\sigma [\varphi /p] \dashv \vdash _{\textbf{L}} \sigma [\psi /p]$ for any $\sigma \in \texttt {Form}(\mathcal {L})$ and propositional variable *p*.

### Odintsov and Wansing’s Criterion of Hyperintensionality

In a recent article Odintsov and Wansing (2021) put forward a criterion for a propositional logic to be hyperintensional based solely on its consequence relation. According to Odintsov and Wansing, a logic is hyperintensional iff its consequence relation is not self-extensional.

#### Definition 2.3

($\textbf{HYP}_L$) A logic $\langle \mathcal {L}, \vdash _{\textbf{L}}\rangle$ is Odintsov-Wansing hyperintensional iff $\vdash _{\textbf{L}}$ is not self-extensional.

Follwing this definition, a logic ${\textbf {L}}$ is hyperintensional iff it has at least one connective or operator for which the following congruence rule is *not* admissible in ${\textbf {L}}$:

$$ \text {if } \varphi _i \dashv \vdash _{{\textbf {L}}} \psi _i \text { for } I =1, \dots , n, \text { then } \#(\varphi _1, \dots , \varphi _n) \dashv \vdash _{{\textbf {L}}} \# (\psi _1, \dots , \psi _n). $$

An example of a hyperintensional logic in this sense is Nelson’s four valued paraconsistent logic ${\textbf {N4}}$ (Almukdad & Nelson, 1984). Indeed, from the following axioms of ${\textbf {N4}}$:

$$\begin{aligned}&\lnot (\varphi \rightarrow \psi ) \leftrightarrow (\varphi \wedge \lnot \psi ) \\&\lnot \lnot \varphi \leftrightarrow \varphi \\&\lnot \varphi \wedge \lnot \psi \rightarrow \lnot (\varphi \vee \psi ) \\&\lnot (\varphi \wedge \psi ) \rightarrow \lnot \varphi \vee \lnot \psi \end{aligned}$$

and the fact that the logic has the deduction Theorem, we obtain that:

$$\begin{aligned} \lnot (\varphi \rightarrow \psi ) \dashv \vdash \lnot (\lnot \varphi \vee \psi ) \text { but not } \lnot \lnot (\varphi \rightarrow \psi ) \dashv \vdash \lnot \lnot (\lnot \varphi \vee \psi ). \end{aligned}$$

This makes the ${\textbf {N4}}$ negation non-congruential, and hence ${\textbf {N4}}$ is not self-extensional. According to Odintsov and Wansing (2021, p. 52), this is simply due to the fact that, whereas the ${\textbf {N4}}$ implication internalises truth preservation, the fact that the ${\textbf {N4}}$ negation does not contrapose implies that the conditional does not internalise falsity preservation as well. Hence, it will not be the case that $\varphi \dashv \vdash _{{\textbf {N4}}} \psi$ iff $\lnot \varphi \dashv \vdash _{{\textbf {N4}}} \lnot \psi$.

Indeed, Odintsov and Wansing note, if self-extensionality were expressed bilaterally,^4^ with logical equivalence formulated in terms of both truth preservation and falsity preservation, ${\textbf {N4}}$ would not be hyperintensional in the $\textbf{HYP}_L$ sense, since it satisfies the following replacement property:

$$ \varphi \dashv \vdash \psi \text { and } \lnot \varphi \dashv \vdash \lnot \psi \text { implies } \sigma [\varphi / p ]\dashv \vdash \sigma [\psi /p]. $$

(Stern, 2025, p. 11-12) states that this phenomenon “forces us to take bilateralism seriously”. Indeed, propositions expressed by formulae can be understood as a tuple of truthmakers and falsemakers for that formula, where truthmakers and falsemakers are not the complement of one another. On this perspective, then, if two formulae are taken to express the same semantic content (i.e., their truthmakers and falsemakers coincide) they will be substitutable salva veritate in every context.^5^

According to the analysis provided by Odintsov and Wansing (and supported by Stern’s claims), it seems that the mark of hyperintensionality is a mismatch between the notion of interderivability given a logic’s consequence relation and sameness of semantic content.^6^

Odintsov and Wansing’s definition of a formal criterion for the hyperintensionality of a logic is formulated with the aim to assess whether Hannes Leitgeb’s logic ${\textbf {HYPE}}$ (Leitgeb, 2019) is hyperintensional.

In particular, Odintsov and Wansing question the significance of some of Leitgeb’s claims regarding ${\textbf {HYPE}}$ and hyperintensionality: “The name ‘${\textbf {HYPE}}$’ might perhaps be seen as suggesting that ${\textbf {HYPE}}$ is a hyperintensional logic creating contexts that ‘do not respect logical equivalence.’ [...] This impression would, however, be wrong” (Odintsov & Wansing, 2021, p. 50). Indeed, as they note, Leitgeb states that “[m]easured by the standards of classical logic and semantics, ${\textbf {HYPE}}$’s *→ * and *↔ * are hyperintensional” whereas “relative to ${\textbf {HYPE}}$’s own standards [...] they are intensional and hence non-extensional” (Leitgeb, 2019, p. 393). Odintsov and Wansing support the latter part of the claim by dubbing ${\textbf {HYPE}}$ as not hyperintensional, based on the self-extensionality of its consequence relation: since ${\textbf {HYPE}}$ is self extensional (Leitgeb, 2019, Observation 20), it is not hyperintensional in the sense of $\textbf{HYP}_L$.

On the other hand, they state that the former part of the claim seems to be misguided: “If this statement is intended to mean that substituting classically logically equivalent formulas within a formula from the language of ${\textbf {HYPE}}$ [...] may result in formulas that fail to be logically equivalent in ${\textbf {HYPE}}$, this is very problematic” (Odintsov & Wansing, 2021, p. 50). This is because, as their argument goes, the meaning of a connective in a given non-classical logic will likely differ from the meaning of the same connective in classical logic. Indeed, “the result of the substitutions would be a formula which has no interpretation in $\textbf{HYPE}$” (Odintsov & Wansing, 2021, p. 50), since the interpretation of the classical connectives is not directly transferrable to $\textbf{HYPE}$. It is then unclear in which sense logics which do not have the same connectives as classical logic create contexts relative to classical logic. They conclude, then, that the most natural way to formulate a criterion of hyperintensionality for a logic is via $\textbf{HYP}_L$.

### Objections to Odintsov and Wansing’s Analysis

A few objections can be moved to this analysis, and to the suggestion to characterise $\textbf{HYP}_L$ as a necessary and sufficient condition for hyperintensionality. $\textbf{HYP}_L$ arguably captures the informal definition of hyperintensionality as failure of intersubstitutivity of logical equivalents from Cresswell (1975, p. 25), also found in Jago (2014, p. 50). As Odintsov and Wansing (2021, p. 50) themselves note, “it seems justified to assume that both Cresswell and Jago have in mind that hyperintensional operators form linguistic contexts in which the substitution of formulas that are *classically* logically equivalent may give rise to non-equivalent formulas”. This is safe to assume, since, arguably, logical equivalence just meant classical logical equivalence for Cresswell, and Cresswell was formulating a definition of hyperintensional contexts directed towards the addition of operators to classical logic.

Based on this interpretation, and on their objection from the meaning of connectives, Odintsov and Wansing conclude that formulating hyperintensionality for a non-classical logic with respect to classical equivalents does not make sense. However, this objection is unlikely to capture the intended interpretation of Leitgeb’s claim: if it did, it would presuppose a rather naïve reading of what it means for a logic to be hyperintensional with respect to classical logic.

Observe, for instance, that if we took the claim that ${\textbf {HYPE}}$ is hyperintensional with respect to classical logic as the failure of the following, for some context *θ *

$$\begin{aligned} \varphi \dashv \vdash _{\textbf{CL}} \psi \ \text {implies} \ \theta (\varphi /p) \dashv \vdash _{{\textbf {H}}} \theta (\psi /p), \end{aligned}$$

this would also entail that almost every non-classical logic is hyperintensional in this sense.^7^ In particular, it would entail that even intuitionistic logic is hyperintensional, since, for example

$$\lnot A \vee A \dashv \vdash _{\textbf{CL}} A \rightarrow A \text { does not imply } \theta (\lnot A \vee A/p) \dashv \vdash _{\textbf{INT}} \theta (A \rightarrow A/p). $$

But it can hardly be said that intuitionistic logic exceeds the resources of intensionality.^8^

Interpreting Cresswell’s notion of hyperintensionality, and Leitgeb’s claims as Odintsov and Wansing seem to do appears to be inaccurate; yet, we are still falling short of a plausible way of generalising the notion of failure of intersubstitutivity salva veritate of logical equivalents to non-classical contexts.

A second, and perhaps more worrisome, point of criticism of Odintsov and Wansing’s treatment of hyperintensionality has to do with the “diagnosis” that seems to emerge from their analysis. As argued above, hyperintensionality understood as failure of self-extensionality can be interpreted as stemming from a mismatch between interderivability in a given logic and sameness of semantic content. Whenever two formulae are interderivable, but the derivability relation is not “symmetrical”, in the sense that interderivability does not correspond to sameness of semantic content, a logic is hyperintensional.

However, it can be seen that the criterion overgenerates, in the sense that it identifies as hyperintensional logics which don’t seem to display any hyperintensional behaviour in this sense. Whenever we consider logics displaying an operator such that the logic inside the operator is not the same as outside the operator, we obtain contexts which are hyperintensional according to $\textbf{HYP}_L$. Indeed, any operator such that the logic inside the scope of the operator differs from the logic outside of it generates a non-self-extensional context.

Yet, it is not clear that such a context would be hyperintensional in the intended sense of the term. In fact, it is ambiguous whether the failure of self-extensionality in this case is equivalent to the operator not respecting the intersubstitutivity salva veritate of logical equivalents; said logical equivalents are not equivalent anymore inside the scope of the operator, the internal and external logic being different. Examples of logics displaying this type of behaviour are, for instance, the modal logics developed by Nicolai and Stern (2021). These logics correspond to the modal logics of various axiomatisations of fixed-point models of truth, such as Kripke-Feferman truth (Feferman, 1991).

Essentially, the necessity operator in these logics translates the truth predicate of the corresponding axiomatic theory of truth. In several axiomatisations of fixed-point models for truth, there is a mismatch between the so-called *internal logic* of the theory, that is, the logic inside the truth predicate, and the external logic, which is simply the logic in which the theory is formulated. For instance, the Kripke-Feferman theory can be understood as a classical axiomatisation of a non-classical fixed-point construction, where the fixed point construction may be formulated in Strong Kleene logic ${\textbf {K3}}$, the Logic of Paradox ${\textbf {LP}}$ or First-Degree Entailment ${\textbf {FDE}}$. The fixed-point construction is carried out in non-classical logic to allow for classically paradoxical sentences to have a gappy, or glutty, truth value, but the axiomatisation, obtained by “closing off” the fixed-point construction, is classical. Then, the corresponding modal logic will present a mismatch between the logic inside the necessity operator and outside. As an example, the logic $\mathbf {M^f}$ presented by Nicolai and Stern has a primitive conditional *↠ * which, outside the necessity operator, collapses to the material conditional, but does not do so inside. Then:

$$ \varphi \supset \psi \dashv \vdash _{\mathbf {M^f}} \varphi \twoheadrightarrow \psi , \text { but not } \Box (\varphi \supset \psi ) \dashv \vdash _{\mathbf {M^f}}\Box (\varphi \twoheadrightarrow \psi ) $$

This corresponds to ${\textbf {M}}^{{\textbf {f}}}$ being non-self-extensional, and hence $\textbf{HYP}_L$ hyperintensional. However, the failure of self-extensionality here is not due to a mismatch between interderivability and sameness of semantic content, as was the case for the ${\textbf {N4}}$ negation. Since the external logic is classical, we also get that $\lnot (\varphi \supset \psi ) \dashv \vdash _{{\textbf {M}}^{{\textbf {f}}}} \lnot (\varphi \twoheadrightarrow \psi )$: then, even if we formulate a congruence criterion where interderivability is expressed bilaterally, the hyperintensional behaviour would persist. In fact, interderivability, both inside and outside the necessity operator, does track sameness of semantic content.^9^

This observation seems to suggest a dilemma: if we take hyperintensionality for logics to just coincide with the failure of self-extensionality, we have to accept that these kinds of modal logics are hyperintensional (and, consequently, that Kripke-Feferman truth is hyperintensional). This seems to generate slightly unintended consequences. In this case, hyperintensionality would not be explained as a mismatch between interderivability and sameness of semantic content, but simply by the fact that the truth (or necessity) operator generates a context governed by a different logic. While, in a sense, this does corresponds to a context not respecting logical equivalence,^10^ this is the case without any mismatch between interderivability and sameness of semantic content, hence it is not clear whether this context would count as hyperintensional in an intended sense. Otherwise, we have to take seriously the option that, while non-self-extensionality captures some hyperintensional logics, it does not seem to be a sufficient condition for hyperintensionality.

In fact, one may argue that it is not even necessary: logics which are “supposed” to be hyperintensional may not satisfy $\textbf{HYP}_L$. For instance, it has been extensively argued that exact truthmaker semantics adequately captures phenomena generating hyperintensional contexts [see for instance (Fine, 2012)]. However, Korbmacher shows that the logic of exact entailment on the so-called replete semantics^11^ is self-extensional, and hence non hyperintensional in the $\textbf{HYP}_L$ sense (Korbmacher, 2023, Corollary 4.9). While the replete semantics adds some “symmetry conditions” with respect to the inclusive semantics, which make it congruential, it is unclear that the failure of self-extensionality would make it inadequate as a framework to model hyperintensional contexts, such as those considered in the several applications of exact truthmaker semantics.

### Williamson’s Criterion

A further argument in favour of the fact that non-self-extensionality is neither necessary nor sufficient for hyperintensionality is given by analysing a definition of hyperintensional context formulated by Timothy Williamson relative to the classical modal logic ${\textbf {S5}}$ (Williamson, 2006). Williamson’s definition suggests the natural formulation of a further hyperintensionality criterion.

Williamson defines self-extensional (or congruential) and non-hyperintensional contexts over classical ${\textbf {S5}}$. His definition of self-extensionality is equivalent, over classical logic, to the one provided in Definition 2.2. A context *θ * is self-extensional iff, for all $\varphi , \psi$:

$$ \vdash (\varphi \equiv \psi ) \text { implies } \vdash (\theta (\varphi /p ) \equiv \theta (\psi /p)). $$

A context *θ * is non-hyperintensional iff, for all $\varphi , \psi$:

$$ \vdash \Box (\varphi \equiv \psi ) \supset \Box (\theta (\varphi /p) \equiv \theta (\psi /p)). $$

Williamson defines hyperintensional contexts as contexts which are not non-hyperintensional. In the interest of comparing Williamson’s definition with Odintsov and Wansing’s characterisation of hyperintensionality, it in necessary to extend his definition of a hyperintensional sentential context in classical logic to logical consequence relations. There are, however, several possible ways to generalise this definition to define a formal criterion for hyperintensionality.

While it is outside the scope of this paper to discuss possible generalisations in detail, I will suggest a few options and briefly discuss them.

Then, I will illustrate an example of divergence between a Williamson-style criterion of hyperintensionality and $\textbf{HYP}_L$.

#### Definition 2.4

Let $\mathcal {L}$ be a language with a necessity modality *\Box * in its signature. Let ${\textbf {L}} =\langle \mathcal {L}, \vdash _{{\textbf {L}}}\rangle$ be a logic for $\mathcal {L}$. Let *→ * and *↔ * indicate, respectively, the conditional and biconditional for ${\textbf {L}}$.^12^ The logic ${\textbf {L}}$ is non-hyperintensional iff for all contexts *θ *, for all $\varphi , \psi$:

1 $$\begin{aligned} \vdash _{{\textbf {L}}} \Box (\varphi \leftrightarrow \psi ) \text { implies } \vdash _{{\textbf {L}}}\Box (\theta (\varphi /p) \leftrightarrow \theta (\psi /p)). \end{aligned}$$

${\textbf {L}}$ is said to be strictly non-hyperintensional iff for all contexts *θ *, for all $\varphi , \psi$:

2 $$\begin{aligned} \vdash _{{\textbf {L}}} \Box (\varphi \leftrightarrow \psi ) \rightarrow \Box (\theta (\varphi /p) \leftrightarrow \theta (\psi /p)). \end{aligned}$$

The criteria just outlined are defined specifically for modal logics. However, Williamson’s criterion can also be extended to logics which don’t feature primitive modal operators, provided that they are embeddable into some modal logic. An embedding from a logic ${\textbf {L}} = \langle \mathcal {L}, \vdash _{\textbf{L}}\rangle$ to a logic ${\textbf {L'}}= \langle \mathcal {L'}, \vdash _{{\textbf {L}}'}\rangle$ is an injective function $f :\texttt {Form}_{\mathcal {L}} \longrightarrow \texttt {Form}_{\mathcal {L'}}$ such that for all $\Gamma \subseteq \texttt {Form}_{\mathcal {L}}$ and $\varphi \in \texttt {Form}_{\mathcal {L}}$, $\Gamma \vdash _{\textbf{L}}\varphi$ iff $\{f(\gamma ) \ | \ \gamma \in \Gamma \} \vdash _{{\textbf {L}}'} f(\varphi )$.

#### Definition 2.5

Let ${\textbf {L}}$ be embeddable into a modal logic ${\textbf {L'}}$. Then, ${\textbf {L}}$ is (strictly) non-hyperintensional iff ${\textbf {L'}}$ is (strictly) non-hyperintensional.

Note that self-extensionality implies Non-Hyp in the presence of necessitation, and it implies Non-Hyp’ in the presence of necessitation and the deduction theorem. Also, all extensional contexts, defined as contexts *θ * satisfying, for all $\varphi , \psi$:

$$ \vdash (\varphi \equiv \psi ) \supset (\theta (\varphi /p) \equiv \theta (\psi /p)), $$

satisfy Non-Hyp’ in the presence of necessitation and the $\text {K}$ axiom. Conversely, in the presence of necessitation, all contexts satisfying Non-Hyp’ (and hence Non-Hyp) are self-extensional.

With these generalisations of Williamson’s notion of non-hyperintensionality at hand, we are ready to formulate a modal criterion of hyperintensionality.

#### Definition 2.6

($\textbf{HYP}_\Box$) A logic $\langle \mathcal {L}, \vdash _{\textbf{L}}\rangle$ is *necessity-hyperintensional* iff

- If ${\textbf {L}}$ is a modal logic $\vdash _{\textbf{L}}$ does not satisfy Non-Hyp.
- If ${\textbf {L}}$ is embeddable into a modal logic ${\textbf {L'}}$, $\vdash _{{\textbf {L'}}}$ does not satisfy Non-Hyp.

#### Definition 2.7

(${\textbf {HYP}}_{\Box }'$) A logic $\langle \mathcal {L}, \vdash _{\textbf{L}}\rangle$ is *strictly necessity-hyperintensional* iff

- If ${\textbf {L}}$ is a modal logic $\vdash _{\textbf{L}}$ does not satisfy Non-Hyp’.
- If ${\textbf {L}}$ is embeddable into a modal logic ${\textbf {L'}}$, $\vdash _{{\textbf {L'}}}$ does not satisfy Non-Hyp’.

The formulation of $\textbf{HYP}_\Box$ and $\textbf{HYP}_\Box '$ crucially depends on the possibility to express necessary equivalence using the conditional in connection with the modal operator. There are logics which are self-extensional without being non-hyperintensional, and viceversa. An example, once again, can be found in Nicolai and Stern’s modal logic ${\textbf {M}}^{{\textbf {f}}}$: as shown in the previous section, ${\textbf {M}}^{{\textbf {f}}}$ satisfies $\textbf{HYP}_L$, but it does not satisfy neither $\textbf{HYP}_\Box$ nor $\textbf{HYP}_\Box '$. Indeed, $\vdash _{\mathbf {M^f}} \Box (\varphi \equiv \psi ) \supset \Box (\theta (\varphi /p) \equiv \theta (\psi /p))$ for all contexts *θ * and for all formulae $\varphi , \psi$. Then, even though according to Odintsov and Wansing logics are hyperintensional only if they are not self-extensional, ${\textbf {M}}^{{\textbf {f}}}$ seems to provide an example of a logic that “shouldn’t” be hyperintensional, even if non-self-extensional. Indeed, ${\textbf {M}}^{{\textbf {f}}}$ is not necessity-hyperintensional. This is explained by the fact that the logic inside the necessity operator is self-extensional, hence necessary equivalents will be intersubstitutable in all contexts.^13^

If we were to apply $\textbf{HYP}_\Box$ to ${\textbf {N4}}$, however, we would obtain a similar result. In fact, ${\textbf {N4}}$ is embeddable into the negation-free fragment of intuitionistic logic (Kamide & Wansing, 2015), which in turn can be faithfully embedded into ${\textbf {S4}}$ (Troelstra & Schwichtenberg, 2000). On this point, Odintsov and Wansing note that “embeddability into a normal modal logic, although it might be seen as suggesting that the translated logic is not hyperintensional, does not imply self-extensionality” (Odintsov & Wansing, 2021, p. 53). Indeed, the embeddability into ${\textbf {S4}}$ of ${\textbf {N4}}$ implies that ${\textbf {N4}}$ is not necessity-hyperintensional.

According to Odintsov and Wansing, “it seems to be uncontroversial that normal modal logics are intensional but not hyperintensional logics” (Odintsov & Wansing, 2021, p. 47). Hence, since ${\textbf {HYPE}}$ is embeddable in a normal bi-modal logic, it should not be hyperintensional. However, this remark seems to contradict what they say about ${\textbf {N4}}$: if normal modal logics are intensional but not hyperintensional, embeddability in a normal modal logic should suggest non-hyperintensionality for ${\textbf {N4}}$ as well. The only thing differentiating the behaviour of ${\textbf {HYPE}}$ from that of ${\textbf {N4}}$ is that, while the former is self-extensional, the latter isn’t. Yet, as the example of ${\textbf {M}}^{{\textbf {f}}}$ shows, non-self-extensionality is not restricted only to logics that intended to capture some mismatch between interderivability and sameness of semantic content.

On the one hand, then, ${\textbf {M}}^{{\textbf {f}}}$ is a “seemingly non-hyperintensional” logic which is non-self-extensional, and its non-hyperintensional character is captured by the fact that it fails to satisfy $\textbf{HYP}_\Box$. On the other hand, ${\textbf {N4}}$ is a logic which does seem, intuitively, to license hyperintensional contexts, and indeed is non-self-extensional, but fails to satisfy necessity-hyperintensionality. This should, at least, provide evidence against the claim that “a logic is hyperintensional only if it is non-self-extensional” (Odintsov & Wansing, 2021, p. 53). I suggest that also the converse direction should be questioned: it is not the case that if a logic is hyperintensional, it is non-self-extensional. We can indeed find examples of congruential hyperintensional logics: they will simply satisfy other criteria for hyperintensionality.

## Fine(r)-Grained Hyperintensionality

### Hyperintensionality and Classical Consequence Relation

Let us go back to defining hyperintensional contexts as contexts which fail to license the intersubstitutivity salva veritate of sentences with the same intension. While co-intensionality can be interpreted as logical equivalence for any given logic, as argued above, it is also possible to specify intensions in terms of classical logical equivalents.

A definition of hyperintensionality in these terms, however, needs some clarification, to avoid incurring in Odintsov and Wansing’s “naïve” interpretation. One way to understand it is suggested by Leitgeb (2019, p. 307): if the set of possible worlds used to specify intensions *coincides with the set of logically possible worlds*, two sentences may be logically equivalent in classical logic without being intersubstitutable salva veritate in hyperintensional contexts. Classical co-intensionals, then, can be understood as sentences which are equivalent at all classical possible worlds, where the notion of “classical possible world” can be accurately specified in a non-classical setting.

In fact, Leitgeb characterises $\textbf{HYPE}$’s conditional as defining hyperintensional contexts *in the standard sense* with respect to classical logic and semantics, but does not make any remarks about the hyperintensionality of $\textbf{HYPE}$’s own consequence relation: “The aim of the semantics would not be to change classical logic per se (as e.g. intuitionists would urge) but to capture the fact that mutual substitution of classically logically equivalent formulas within S-contexts^14^ should not preserve truth with logical necessity” (Leitgeb, 2019, p. 311).

Leitgeb’s intended interpretation of the hyperintensionality of ${\textbf {HYPE}}$ with respect to classical equivalents is then based on a semantic understanding. ${\textbf {HYPE}}$ is based on a situation semantics, that is, a semantics allowing both for partial and overdetermined worlds (see Barwise & Perry, 1983, ch. 3); further, it features elements from possibility semantics, such as monotonicity constraints (see Humberstone, 1981). Both situation semantics and possibility semantics are generalisations of possible worlds semantics, that do not require states^15^ to obey the laws of classical logic. However, by imposing further constraints on the behaviour of states, these semantic frameworks can recover classical logic. Then, classical possible worlds are a subset of the space of situations, or the space of possibilities.

From a semantic point of view, then, hyperintensionality with respect to the classical consequence relation can be defined, in a logic with a state-based semantics, as the failure of intersubstitutivity of formulae which are equivalent at all classical possible worlds. This is, indeed, exactly the sense in which Korbmacher understands hyperintensionality as failure of intersubstitutivity for classical equivalents (Korbmacher, 2023, p. 15). Korbmacher’s analysis of the logic of exact entailment as hyperintensional with respect to classical equivalents is not susceptible to the same “naïve reading” objection as Odintsov and Wansing’s critique of ${\textbf {HYPE}}$, exactly because Korbmacher’s analysis is based on semantical entailment.

Based on this reasoning, then, the successful formulation of a formal criterion for hyperintensionality with respect to classical equivalents, henceforth CL-hyperintensionality, depends on the possibility to accurately capture this semantic understanding.

A natural suggestion would be to consider the so-called recapture theorems which have been formulated for some non-classical logics, as presented for example by Beall (2013). Beall shows that, for some logics, such as ${\textbf {K3}}$,

$$\text {If } \Gamma \vdash _{\textbf {CL}}\Delta \text { then } e(\Delta ) \cup \Gamma \vdash _{{\textbf {K3}}} \Delta , $$

where *e(Δ )* is a so-called “completeness set” for *Δ *, that is, a set of formulae of the form $p \vee \lnot p$ for all propositional variables *p* in *Δ *. Since ${\textbf {K3}}$ does not have excluded middle, what recapture essentially says is that we can recover classical derivability in ${\textbf {K3}}$ by only adding the instances of excluded middle that we need. Intuitively, the notion of a state semantics which can recover the meaning of classical connectives at classical possible worlds should be captured by these recapture claims.

A tentative formulation of hyperintensionality in these terms would then be the following. Let a logic ${\textbf {L}}$ have a recapture theorem, such that, for some “recapture set” *e(φ )*:

$$\text {If } \Gamma \vdash _{\textbf {CL}}\varphi \text { then } e(\varphi ) \cup \Gamma \vdash _{{\textbf {L}}} \varphi . $$

Then, ${\textbf {L}}$ is CL-hyperintensional iff it is not the case that for all $\varphi , \psi , \theta$ and all propositional variables *p*, $\varphi \dashv \vdash _{\textbf {CL}}\psi$ implies $\theta (\varphi /p) \cup e(\psi ) \dashv \vdash _{{\textbf {L}}} \theta (\psi /p) \cup e(\varphi )$.

Consider, once again, the case of ${\textbf {HYPE}}$. ${\textbf {HYPE}}$ satisfies the following (Leitgeb, 2019, Observation 40):

$$\begin{aligned} A \vdash _{\textbf {CL}}B \text { iff } A \cup \Phi \vdash _{\text {H}}B \end{aligned}$$

where $$\Phi = \{ A \vee \lnot A \vert A \in \mathtt{Form} \} \cup \{D[A \rightarrow C] \leftrightarrow D[\lnot A \vee C] \vert A,C \in \mathtt{Form}\}$$.^16^

Hence, models of classical logic can be seen as special cases of $\textbf{HYPE}$-models, in which the $\textbf{HYPE}$ negation coincides with classical negation, and $\textbf{HYPE}$’s conditional coincides with the material conditional (Leitgeb, 2019).^17^${\textbf {HYPE}}$ would then be CL-hyperintensional iff it is not the case that for all $\varphi , \psi , \theta$ and all propositional variables *p*, $\varphi \dashv \vdash _{\textbf {CL}}\psi$ implies $\theta (\varphi /p) \cup \Phi \dashv \vdash _{\textbf {H}}\theta (\psi /p) \cup \Phi$. This is clearly not the case for ${\textbf {HYPE}}$ (Leitgeb, 2019, Observation 40).    

However, the formulation of hyperintensionality with respect to classical logic in terms of recapture results poses some problems. First of all, this understanding of hyperintensionality depends on specific ways of interpreting the logic at issue, because it is only applicable in contexts in which we can add suitable recapture sets to recover the meaning of classical connectives.^18^ However. it would be desirable to have a criterion of hyperintensionality with respect to classical logic that is independent of changes in the semantics of the logic at issue. In this case, a syntactic approach sounds more plausible; but care must be taken not to incur in Odintsov and Wansing’s criticism.^19^

A possible way of capturing the idea of recovering the meaning of classical connectives is to consider cases in which there is a translation between the logic under consideration and classical logic preserving the meaning of connectives. To achieve this aim, such a translation should be fixed and faithful. Then, the meaning of connectives is the same between the logic $\textbf{L}$ under consideration and classical logic iff the logic satisfies the following:

3 $$\begin{aligned} \vdash _{\textbf{CL}} \varphi \text{ iff } \vdash _{\textbf{L}} \varphi ^{L}, \text { for } L \text{ a fixed and faithful translation.} \end{aligned}$$

Failure of self-extensionality with respect to classical logic can be formulated as:

4 $$\begin{aligned} \varphi \dashv \vdash _{\textbf {CL}}\psi \text { fails to imply } \theta (\varphi ^L/p) \dashv \vdash _{\textbf{L}} \theta (\psi ^L/p) \end{aligned}$$

Hence, CL-hyperintensionality is defined as follows:

#### Definition 3.1

($\mathbf {HYP_{CL}}$) A logic $\langle \mathcal {L}, \vdash _{\textbf{L}}\rangle$ such that TC holds for $\vdash _{\textbf{L}}$ is CL-hyperintensional iff it satisfies N-Congr-CL.

In order to assess this criterion, let us once again evaluate it against the backdrop of ${\textbf {HYPE}}$: as it turns out, even according to ${\textbf {HYP}}_{{\textbf {CL}}}$, ${\textbf {HYPE}}$ is not hyperintensional with respect to classical logic.

Because of the relationship between $\textbf{HYPE}$ and intuitionistic logic, results about intuitionistic logic can be translated into $\textbf{HYPE}$ by adapting the standard double negation translation to the $\textbf{HYPE}$ setting. We can thus obtain a fixed and faithful translation between classical logic and $\textbf{HYPE}$. Since $\lnot \varphi \dashv \vdash _{\textbf {INT}}\varphi \rightarrow \bot$, whereas the equivalence clearly does not hold in $\textbf{HYPE}$, we can define a “double negation translation” (which we will call *H*-translation) for $\textbf{HYPE}$ as follows:

$$\begin{aligned}&\varphi ^H = (\varphi \rightarrow \bot ) \rightarrow \bot \text { for } \varphi \in \texttt {Prop}\\&(\lnot \varphi )^H = \varphi ^H \rightarrow \bot \\&(\varphi \wedge \psi )^H = \varphi ^H \wedge \psi ^H\\&(\varphi \vee \psi )^H = ((\varphi ^H \rightarrow \bot ) \wedge (\psi ^H \rightarrow \bot )) \rightarrow \bot \\&(\varphi \rightarrow \psi )^H = \varphi ^H \rightarrow \psi ^H \end{aligned}$$

#### Lemma 3.2

$$\vdash _{\textbf {CL}}\varphi \textrm{ iff } \vdash _{\textbf {H}}\varphi ^H$$.    

#### Proof

Left to right. It is sufficient to verify this for all axioms and rules of classical logic presented in the usual Hilbert-style calculus. This is easily done by recalling the usual Gödel-Gentzen translation for intuitionistic logic (*N*-translation):

$$\begin{aligned}&\varphi ^N = \lnot \lnot \varphi \text { for } \varphi \in \texttt {Prop}\\&(\lnot \varphi )^N = \lnot \varphi ^N \\&(\varphi \wedge \psi )^N = \varphi ^N \wedge \psi ^N\\&(\varphi \vee \psi )^N = \lnot (\lnot \varphi ^N \wedge \lnot \psi ^N) \\&(\varphi \rightarrow \psi )^N = \varphi ^N \rightarrow \psi ^N \end{aligned}$$

Since $\lnot \varphi \dashv \vdash _{\textbf {INT}}\varphi \rightarrow \bot$ the *N*-translation is equivalent to the *H*-translation in intuitionistic logic. Hence, for all $\Gamma , \varphi$, $\Gamma \vdash _{\textbf {CL}}\varphi$ implies $\Gamma ^H \vdash _{\textbf {INT}}\varphi ^H$. Since the *H*-translation is *¬ *-free, by Observation 41 in Leitgeb (2019), $\Gamma ^H \vdash _{\textbf {INT}}\varphi ^H$ implies $\Gamma ^H \vdash _{\textbf {H}}\varphi ^H$, hence $\Gamma \vdash _{\textbf {CL}}\varphi$ implies $\Gamma ^H \vdash _{\textbf {H}}\varphi ^H$. The translation *H* is shown to be faithful by a standard proof using classical $\textbf{HYPE}$-models. Assume $\nvdash _{\textbf{CL}} \varphi$. Then there exists a model of classical logic $(D, I, v_j)$ such that $(D, I, v_j) \nvDash \varphi$. But then one can construct a classical $\textbf{HYPE}$-model $\mathfrak {M}$ such that $\mathfrak {M}, j \nVdash \varphi$. By Observation 40 in Leitgeb (2019), if $\mathfrak {M}$ is a classical $\textbf{HYPE}$-model, then $\mathfrak {M}, j \Vdash \varphi$ iff $\mathfrak {M}, j \Vdash \varphi ^H$. Hence, $\mathfrak {M}, j \nVdash \varphi ^H$, so $\nvdash _{\textbf{HYPE}} \varphi ^H$.    

Right to left. $\vdash _{{\textbf {HYPE}}} \varphi ^H$ implies $\vdash _{\textbf {CL}}\varphi$ because the axioms of $\textbf{HYPE}$ are a subset of the axioms of classical logic. *□ *

So there is an equivalence between provability in $\textbf{HYPE}$ and provability in classical logic.

#### Corollary 3.3

If $$A \dashv \vdash _{\textbf {CL}}B$$ then $$A^H \dashv \vdash _{\textbf {H}}B^H$$.

#### Lemma 3.4

For any $\varphi , \psi \in \mathcal {L}_{\textbf{CL}}$, if $\varphi \dashv \vdash _{\textbf {CL}}\psi$ then $\theta (\varphi ^H/p) \dashv \vdash _{\textbf {H}}\theta (\psi ^H/p)$. Therefore $\textbf{HYPE}$ does not satisfy $\mathbf {HYP_{CL}}$.    

#### Proof

This follows from Corollary 3.3 and the fact that $\textbf{HYPE}$ satisfies self-extensionality. *□ *

If two formulae are equivalent in classical logic they will be intersubstitutable in the classical fragment of $\textbf{HYPE}$, and if two formulae are equivalent in classical logic, since this entails that their translations will be equivalent in $\textbf{HYPE}$, they will be intersubstitutable in $\textbf{HYPE}$. Hence, ultimately, ${\textbf {HYPE}}$ is only hyperintensional with respect to classical equivalents if we read hyperintensionality with respect to classical logic in the “naïve” way.

On the other hand, an example of a logic which does satisfy this criterion is ${\textbf {N3}}$. Indeed, the *H* translation works as a fixed and faithful translation for ${\textbf {N3}}$ too: however, since ${\textbf {N3}}$ is non-self-extensional, it will satisfy ${\textbf {HYP}}_{{\textbf {CL}}}$. In fact, ${\textbf {HYP}}_{{\textbf {CL}}}$ is reducible to the failure of extensionality, plus an additional condition stating that the meaning of classical connectives should be recoverable in the system under consideration.

### Weak Hyperintensionality

It seems natural to ask, then, whether there is indeed a sense in which we can vindicate Leitgeb’s claim that ${\textbf {HYPE}}$ is a hyperintensional logic. Korbmacher argues that, since ${\textbf {HYPE}}$, meant as a logical framework for inexact truthmaking, does not satisfy ${\textbf {HYP}}_L$, we should take this as evidence in favour of the fact that we should consider exact truthmaking instead of inexact truthmaking as the privileged semantical framework for the construction of a hyperintensional logic (Korbmacher, 2023, p. 15). A further argument in favour of this view is given by Fine, who maintains that, since inexact truthmaker semantics can be derived from exact truthmaker semantics the latter should be taken as the basis of a privileged system of hyperintensional logic (Fine, 2017).

However, as noted by Deigan (2020) and Leitgeb (2021), the converse can be done as well. In particular, Leitgeb shows that we can define exact truthmaker semantics from $\textbf{HYPE}$ by adding totality facts. Since the logic of exact entailment, which is the logical framework for exact truthmaking, satisfies ${\textbf {HYP}}_L$, and since the addition of totality facts only singles out a special case of inexact truthmaking, it would be very suspicious if $\textbf{HYPE}$ weren’t hyperintensional *at all*. Also, Korbmacher notes, “[Fine’s] system for exact entailment on the replete semantics,^20^ [...] turns out not to be hyperintensional in the sense of Odintsov and Wansing” (Korbmacher, 2023, p. 3). There are special cases of systems of exact truthmaker semantics which give rise to logics failing to be Odintsov-Wansing hyperintensional; the appropriate reaction to this should be, in my opinion, to understand what notion of hyperintensionality underlies logics for inexact truthmaker semantics, instead of dismissing them as not hyperintensional *tout court*. Note, for instance, that Linnebo’s intrinsic truthmaking framework (Linnebo, 2022) would fail to be hyperintensional on the Odintsov-Wansing criterion, while we would expect it to be hyperintensional.

The hyperintensional contexts which can arise against the background of ${\textbf {HYPE}}$ fail to be captured by the criteria outlined above. However, there is a way to characterise ${\textbf {HYPE}}$ and logics which share some characteristics with it as more than intensional, and hence, to a certain extent, hyperintensional. As discussed in the introduction, specifying a criterion for hyperintensionality ultimately depends on how we interpret sameness of intension in a given setting. Instead of being specified with respect to classical possible worlds, co-intensionals can be specified with respect to the logical-semantical background framework under considerations, i.e. with respect to states, situations, or possibilities.

There are a couple of possible ways to specify sameness of intension in this sense. A first option is to define hyperintensional contexts as context which do not license the intersubstitutivity salva veritate of formulae which are true at the same set of states, situations or possibilities (instead of being true at the same set of possible worlds) in a given model. Indeed, Leitgeb defines two sentences to have the same intension in ${\textbf {HYPE}}$ when they have the same extension at each state (Leitgeb, 2019, p. 307). Obviously, this restricts the applicability of the criterion to logics that can be represented by a state-based semantics, or, equivalently, by some kind of intensional semantics. A definition in these terms can be spelled out thus:

#### Definition 3.5

($\textbf{HYP}_{\texttt {int}}$) Let a logic ${\textbf {L}} = \langle \mathcal {L}, \vdash _{\textbf{L}}\rangle$ be sound and complete with respect to a class of frame structures *A*. Let *X ∈ A* and let $\mathfrak {M}: = \langle X, v\rangle$ be any model of ${\textbf {L}}$. ${\textbf {L}}$ is ${\textbf {HYP}}_{\texttt {int}}$-hyperintensional iff the following condition holds for all $\varphi , \psi \in \texttt {Form}$ and for all contexts *θ *.

For all states *s ∈ X*: $\mathfrak {M}, s \vDash \varphi \leftrightarrow \psi$ does not imply that, for all *s ∈ X*, $\mathfrak {M}, s \vDash \theta (\varphi /p) \leftrightarrow \theta (\psi /p)$.

According to this definition, sameness of intension as truth at the same set of states is captured by the truth of the biconditional at each state of any given model. As it turns out, not even this criterion is successfully applicable to ${\textbf {HYPE}}$.

#### Observation 3.1

${\textbf {HYPE}}$ does not satisfy $\textbf{HYP}_{\texttt {int}}$.

#### Proof

It is sufficient to show that it is impossible, for any given ${\textbf {HYPE}}$-model $\mathfrak {M} = \langle \mathcal {F}, v\rangle$, where $\mathcal {F} = \langle W, \le , *\rangle$ is an involutive Routley frame, to be such that $\forall s \in W: \mathfrak {M}, s \Vdash \varphi \leftrightarrow \psi$ and for it to be the case that there is a *s' ∈ W* such that $\mathfrak {M}, s'\nVdash \theta (\varphi /p) \leftrightarrow \theta (\psi /p)$. We prove this by induction on the length of formulae. The only salient case is that of negation. The semantic clause for negation in ${\textbf {HYPE}}$ is: $\mathfrak {M}, s\Vdash \lnot \varphi$ iff $\mathfrak {M}, s^* \nVdash \varphi$. Assume $\forall s \in W: \mathfrak {M}, s \Vdash \varphi \leftrightarrow \psi$ and $\mathfrak {M}, s'\nVdash \lnot \varphi \leftrightarrow \lnot \psi$. Then, without loss of generality, $\exists s'' \ge s'. \mathfrak {M}, s'' \Vdash \lnot \varphi$ and $\mathfrak {M}, s'' \nVdash \lnot \psi$. Then, $\mathfrak {M}, s''^* \nVdash \varphi$ and $\mathfrak {M}^*, s'' \Vdash \psi$, contradicting the assumption that $\forall s \in W: \mathfrak {M}, s \Vdash \varphi \leftrightarrow \psi$. *□ *

There is, however, an even weaker way to spell out sameness of intension for logics with a state-based semantics. As we saw earlier, the formulation of our different criteria is heavily dependent on the presence of a conditional in the logic we are considering. If we are considering logics with an intensional conditional, i.e a conditional which is evaluated across states, as is the case for ${\textbf {HYPE}}$, sameness of intension can be captured simply by the biconditional, instead of the biconditional holding at each state (which, on the other hand, captures sameness of semantic content). Indeed, truth of the ${\textbf {HYPE}}$ biconditional $\varphi \leftrightarrow \psi$ at a given state indicates that *φ * and *ψ * are true at the same set of accessible states. Hence, for logics with intensional conditionals, we can formulate a further, even weaker criterion of hyperintensionality.^21^

#### Definition 3.6

($\textbf{HYP}_{\texttt {adm}}$) Let a logic ${\textbf {L}} = \langle \mathcal {L}, \vdash _{\textbf{L}}\rangle$ be sound and complete with respect to a class of frame structures *A*. Let *X ∈ A* and let $\mathfrak {M}: = \langle X, v\rangle$ be any model of ${\textbf {L}}$. ${\textbf {L}}$ is admissibly hyperintensional iff the following condition holds for all $\varphi , \psi \in \texttt {Form}$ and for all contexts *θ *.

For all states *s ∈ X*:( $\mathfrak {M}, s \vDash \varphi \leftrightarrow \psi$ does not imply that $\mathfrak {M}, s \vDash \theta (\varphi /p) \leftrightarrow \theta (\psi /p)$.)

#### Observation 3.2

$${\textbf {HYPE}}$$ satisfies $${\textbf {HYP}}_{\texttt {adm}}$$.

#### Proof

This is due to the fact that ${\textbf {HYPE}}$ presents local failures of contraposition (see Leitgeb, 2019, Example 2).^22^ An example can be constructed as follows [using the semantics for ${\textbf {HYPE}}$ introduced by Speranski (2021)]. Let $\mathfrak {M}$ be a ${\textbf {HYPE}}$-model with state space $X = \{s, s^*\}$ such that $s = \{\varphi , \psi \}$, $s^* =\{ \varphi \}$. Then, $\mathfrak {M}, s \Vdash \varphi , \mathfrak {M}, s \nVdash \lnot \varphi , \mathfrak {M}, s \Vdash \psi , \mathfrak {M}, s\Vdash \lnot \psi$, while $\mathfrak {M}, s^* \Vdash \varphi , \mathfrak {M}, s^* \nVdash \lnot \varphi , \mathfrak {M}, s^* \nVdash \psi , \mathfrak {M}, s^*, \nVdash \lnot \psi$. Then, $\mathfrak {M}, s\Vdash \varphi \leftrightarrow \psi$, while $\mathfrak {M}, s \nVdash \lnot \varphi \leftrightarrow \lnot \psi$. Hence, ${\textbf {HYPE}}$ satisfies ${\textbf {HYP}}_{\texttt {adm}}$. *□ *

We can then say that, in a sense, ${\textbf {HYPE}}$’s conditional not only “creates hyperintensional contexts with respect to classical semantics” in the “naïve” sense specified above, but also creates in a very weak sense hyperintensional contexts with respect to ${\textbf {HYPE}}$’s own semantics, albeit not creating hyperintensional contexts relative to its own consequence relation.

## A Case Study

In the previous sections, I presented some remarks which were meant to motivate the formulation of several criteria for hyperintensionality, rooted in different understandings of what it means for a logic to license hyperintensional contexts. The driving dialectic for my analysis was that Odintsov and Wansing’s criterion based on the failure of self-extensionality is neither necessary nor sufficient to accurately characterise hyperintensionality. This is because it both overgenerates, labeling as hyperintensional logics which are not meant to capture any form of hyperintensional behaviour, and undergenerates, failing to recognise as hyperintensional logics which are normally understood to license hyperintensional contexts.

Furthermore, I suggest to take the multiplicity of “informal” definitions of hyperintensional contexts at face value, and to recognise that, when adapted to the context of non-classical logics, they may all capture different nuances of what it means to be hyperintensional.

In this section, I will take stock of these observations, and suggest a way to interpret them. In particular, I will assess against some of the criteria presented above a group of logics, all of which some form of intensional semantics, as well as an interplay between the conditional and the negation. The aim of this analysis will be to show the differences between hyperintensionality criteria and try to gesture towards an interpretation of what it may mean for a logic to be hyperintensional. In particular, I aim to improve and expand upon Stern’s and Odintsov and Wansing’s diagnoses of hyperintensionality in terms of mismatch between interderivability and sameness of semantic content.

While I do not expect to provide a full and definitive diagnosis of what hyperintensionality amounts to, I will highlight a correlation between the satisfaction of different criteria for hyperintensionality (at least for the logics under consideration) and different ways in which forms of contraposition can fail in these logics.

I will firstly introduce the logics I will be considering, both axiomatically and semantically, and explain the salient features of the behaviour of their negation and conditional. Then, I will show which logics satisfy which hyperintensionality criteria, and briefly analyse the results obtained.

### Logics

I will consider three subfamilies of logics: the family of Došen-style logics (Došen, 1986), among which figures ${\textbf {HYPE}}$ [see Speranski (2022) for a unified presentation], the Nelson-style logics ${\textbf {N3}}$ and ${\textbf {N4}}$ [see Latkin (2010)] and the logic ${\textbf {NB}}$ introduced by Avron and Zamansky (2020), which belongs to the family of LFIs, or logics of formal inconsistency [see Carnielli et al. (2007)].

Došen-style logics and Nelson-style logics are built over the negation-free fragment of intuitionistic logic, while ${\textbf {NB}}$ is built over the negation-free fragment of classical logic. In Došen-style logics and in ${\textbf {NB}}$ the negation is defined relative to a frame accessibility relation *R*. In particular, the negation in Došen-style logic and in ${\textbf {NB}}$ can be understood in terms of the framework for negation suggested by Vakarelov (1989).^23^ Negation in Došen-style logics can be understood as a normal impossibility modality, whereas in ${\textbf {NB}}$ negation is understood as a modality of unnecessity. On the other hand, the negation in Nelson-style logics is similar to a constructive negation. It is defined locally, but it interacts with the intensional intuitionistic conditional.

#### Definition 4.1

The negation-free fragment of intuitionistic logic ${\textbf {IL}}^+$ is the system obtained by closing the following axiom schemata under modus ponens.

1. $$\psi \rightarrow (\varphi \rightarrow \psi )$$
2. $$(\psi \rightarrow (\varphi \rightarrow \chi )) \rightarrow ((\psi \rightarrow \varphi ) \rightarrow (\psi \rightarrow \chi ))$$
3. $$\psi \wedge \varphi \rightarrow \psi $$, $$\psi \wedge \varphi \rightarrow \varphi $$
4. $$\psi \rightarrow (\varphi \rightarrow (\varphi \wedge \psi ))$$
5. $$\psi \rightarrow \psi \vee \varphi $$, $$\varphi \rightarrow \psi \vee \varphi $$
6. $$(\psi \rightarrow \chi ) \rightarrow ((\varphi \rightarrow \chi ) \rightarrow (\varphi \vee \psi \rightarrow \chi )) $$

The negation-free fragment of classical logic ${\textbf {CL}}^+$ is the system obtained by adding to the axioms of ${\textbf {IL}}^+$ the following axiom:

1. $$((\psi \rightarrow \varphi ) \rightarrow \psi ) \rightarrow \psi $$

The system $\textbf{CL}^+_\bot$ is obtained by closing the axioms of $\textbf{CL}^+$, formulated in $\mathcal {L}_\bot$ and the following:

$$\begin{aligned} \bot \rightarrow \varphi \end{aligned}$$

under modus ponens.

Consider the follwing negation axioms and rules:

5 $$\begin{aligned}&\lnot \varphi \vee \varphi \end{aligned} $$

6 $$\begin{aligned}&\lnot \varphi \wedge \lnot \psi \rightarrow \lnot (\varphi \vee \psi ) \end{aligned}$$

7 $$\begin{aligned}&\lnot (\varphi \rightarrow \varphi ) \rightarrow \psi \end{aligned}$$

8 $$\begin{aligned}&\lnot \lnot (\varphi \rightarrow \varphi ) \end{aligned}$$

9 $$\begin{aligned}&\lnot (\varphi \wedge \psi ) \rightarrow \lnot \varphi \vee \lnot \psi \end{aligned}$$

10 $$\begin{aligned}&\varphi \rightarrow \lnot \lnot \varphi \end{aligned}$$

11 $$\begin{aligned}&\lnot \lnot \varphi \rightarrow \varphi \end{aligned}$$

$$\begin{aligned} \text {Ctr}\frac{\varphi \rightarrow \psi }{\lnot \psi \rightarrow \lnot \varphi } \end{aligned}$$

#### Proposition 4.2

(Avron & Zamansky, 2020) Every axiomatic extension of $\textbf{IL}^+$ has the following properties:

- $$\Gamma \vdash _{\textbf{L}} \varphi \rightarrow \psi $$ iff $$\Gamma , \varphi \vdash _{\textbf{L}}\psi $$;
- $$\Gamma \vdash _{\textbf{L}}\varphi \wedge \psi $$ iff $$\Gamma \vdash _{\textbf{L}}\varphi $$ and $$\Gamma \vdash _{\textbf{L}}\psi $$;
- $$\Gamma , \varphi \vee \psi \vdash _{\textbf{L}}\chi $$ iff $$\Gamma , \varphi \vdash _{\textbf{L}}\chi $$ and $$\Gamma , \psi \vdash _{\textbf{L}}\chi $$.

#### Definition 4.3

Došen’s logic $\textbf{N}$ is obtained by closing the axioms of $\textbf{IL}^+$ and DM1 under MP and Ctr (Došen, 1986).

A family of extensions of ${\textbf {N}}$ is presented and investigated by Speranski (2022). We recall the definitions of the so-called Došen-style logics below.

#### Definition 4.4

$$\begin{aligned}&\textbf{N}^\circ := \textbf{N} + \{Expl, DN\}\\&\textbf{N}^* := \textbf{N}^\circ + \{DM2\}\\&\textbf{N}^*_i : = \mathbf {N^*} + \{I1, I2\} \end{aligned}$$

${\textbf {N}}^{\circ }$ can be thought of as the least logic in which *⊥ * behaves like the falsum (Speranski, 2021). ${\textbf {N}}^*$ was introduced in Cabalar (2006). As shown by Speranski (2021), $\textbf{N}^*_i$ is equivalent to Leitgeb’s ${\textbf {HYPE}}$. As noted by Speranski (2022) although the rule Ctr is admissible in ${\textbf {N}}$ and its extensions, it is not derivable.

Next, we consider the so-called Nelson-style logics. The logic ${\textbf {N4}}$ was first presented by Almukdad and Nelson (1984), while the logic ${\textbf {N3}}$ is introduced by Thomason (1969). A comprehensive treatment of these logics can be found in Latkin (2010), Kamide and Wansing (2015) and Odintsov (2003).

#### Definition 4.5

The logic $\textbf{N4}$ is obtained by closing the axiom schemata of $\textbf{IL}^+$, I1, I2, DM1, DM2 and the following axiom:

12 $$\begin{aligned} \lnot (\varphi \rightarrow \psi ) \leftrightarrow (\varphi \wedge \lnot \psi ) \end{aligned}$$

under modus ponens.

Note that the presence of the axiom N4 is inconsistent with contraposition, hence Ctr cannot be a rule in the logic.

#### Definition 4.6

The logic ${\textbf {N3}}$ is obtained by closing the below axiom schemata under modus ponens.

$$\begin{aligned} \textbf{N3}:= \textbf{N4} + Expl. \end{aligned}$$

The logic ${\textbf {NB}}$, on the other hand, is obtained as an axiomatic extension of the negation-free fragment of classical logic, and was introduced by Avron and Zamansky (2020).

#### Definition 4.7

The logic ${\textbf {NB}}$ is obtained by closing the following axioms under the rules of modus ponens and Ctr.

$$ \textbf{NB} := \textbf{CL}^+ + LEM + I2 $$

### Semantics

I will now present the semantics for the logics defined above in terms of frame semantics. I will, where possible, present these logics in terms of a unified semantical framework.

#### Definition 4.8

A frame is a triple $\mathfrak {F} = \langle W, \le ,R\rangle$, with *W* a nonempty set of states, *≤ * a preordering on *W* and *R* a binary relation on *W*, antimonotone with respect to *≤ *. Truth and falsity valuation mappings $v^+, v^-: \texttt {Prop} \longrightarrow W$ assign propositional variables to states in *W*. If, instead of $v^+, v^-$, a single valuation function $v :\texttt {Prop} \longrightarrow W$ is used, it is to be understood as $v = v^+$ and $v^-: = \texttt {Prop} \backslash v^+$. A frame model $\mathfrak {M} = \langle \mathfrak {F}, v^+, v^-\rangle$ is given by a frame and valuation mappings.

We will sometimes abbreviate *(u,v) ∈ R*, with *u,v ∈ W*, as *uRv*. The basic clauses for positive truth at a frame can now be defined. These are common to all the logics we will be considering in the following, while semantic clauses for the negation and implication characterise the behaviour of each logic.

#### Definition 4.9

[Basic (extensional) truth in a frame] Let $\mathfrak {M} = \langle \mathfrak {F}, v\rangle$ be a frame model for $\mathcal {L}_\bot$, such that $v = v^+$ and $v^-: = \texttt {Prop} \backslash v^+$. For every *s ∈ W*, we define $\mathfrak {M}, s \Vdash \varphi$ (*φ * is true at *s* in $\mathfrak {M}$) inductively as follows.

- $$\mathfrak {M}, s \nVdash \bot $$;
- $$\mathfrak {M}, s \Vdash p \text { iff } s \in v(p)$$;
- $$\mathfrak {M}, s \Vdash \varphi \wedge \psi \text { iff } \mathfrak {M}, s \Vdash \varphi \text { and } \mathfrak {M}, s \Vdash \psi $$;
- $$\mathfrak {M}, s \Vdash \varphi \vee \psi \text { iff } \mathfrak {M}, s \Vdash \varphi \text { or } \mathfrak {M}, s \Vdash \psi $$.

I will now recall the negation and conditional clauses for, respectively, Došen-style logics, Nelson-style logics and ${\textbf {NB}}$.

#### Definition 4.10

(Truth in a Došen-style frame) Let $\mathfrak {M} = \langle \mathfrak {F}, v\rangle$ be a frame model for $\mathcal {L}_\bot$, such that $v = v^+$ and $v^-: = \texttt {Prop} \backslash v^+$. Let $R(w): = \{ u \in W \ | \ wRu\}$, so *v ∈ R(w)* iff *wRv*. For every *s ∈ W*, we define $\mathfrak {M}, s \Vdash \varphi$ as supplementing the basic inductive clauses with the following:

- $$\mathfrak {M}, s \Vdash \varphi \rightarrow \psi \text { iff } \forall y \in W, \text { s.th. } s \le y, \text { if } \mathfrak {M}, y \Vdash \varphi \text { then }\mathfrak {M}, y \Vdash \psi $$;
- $$\mathfrak {M}, w \Vdash \lnot \varphi \text { iff } \mathfrak {M}, v \nVdash \varphi $$ for all *v ∈ R(w)*.

#### Observation 4.1

$$\mathfrak {M}, w \Vdash \varphi $$ and $$w \le v \ \ \Longrightarrow \ \ \mathfrak {M}, v \Vdash \varphi $$.

The different Došen style logics are characterised by different properties of the relation *R* (Speranski, 2022).

- $\mathbf {N^\circ }$: let *R* be serial, i.e., $R(w) \ne \varnothing$ for each *w ∈ W*.
- $\mathbf {N^*}$: let *R* be directed with respect to *≤ *, i.e. for every *u,v ∈ W*, there exists *w ∈ W* such that *u ≤ w* and *v ≤ w*. Define an antimonotone function * :W⟶ W picking out the greatest element of *R* and let $\mathfrak {F} = \langle W, \le , * \rangle$. Then let $\mathfrak {M}= \langle \mathfrak {F}, v\rangle$. Truth is defined as in the basic definition for Došen-style logics, letting R = *.
  $$ \mathfrak {M}, w \Vdash \lnot \varphi : \Longleftrightarrow \mathfrak {M}, w^* \nVdash \varphi $$
- $$\mathbf {N^*_i}$$: Let * be involutive, i.e., $$w^{**} = w$$.

#### Definition 4.11

(Nelson-style model) Let $\mathfrak {F}= \langle W, \le \rangle$ be a frame, defined as above, but such that *R = ∅ *. Let $v^+, v^-: \texttt {Prop} \longrightarrow W$ be valuation mappings for truth preservation and falsity preservation defined as above. A Nelson-style model is a tuple $\mathfrak {M} = \langle \mathfrak {F}, v^+, v^-\rangle$. A Nelson-style model is a ${\textbf {N3}}$ model iff $v^+(p) \cap v^-(p) = \varnothing$ for all $p \in \texttt {Prop}$.

If we only have one valuation *v* tracking the preservation of truth in $\textbf{N4}$ or ${\textbf {N3}}$, the frame model generated by this valuation is said to be an *intuitionistic model* of $\textbf{N4}$ or $\textbf{N3}$ (Latkin, 2010).

#### Definition 4.12

(Truth in a Nelson-style model) Let $\mathfrak {M} = \langle \mathfrak {F}, v^+, v^-\rangle$ be a Nelson-style model for $\mathcal {L}_\bot$. Then, for every *s ∈ W*, we define $\mathfrak {M}, s \Vdash ^+ \varphi$ and $\mathfrak {M}, s \Vdash ^- \varphi$ inductively as follows:

1. $$\mathfrak {M}, s \nVdash ^+ \bot $$, $$\mathfrak {M}, s \Vdash ^- \bot $$;
2. $$\mathfrak {M}, s \Vdash ^+ p \text { iff } s \in v^+(p)$$, $$\mathfrak {M}, s \Vdash ^- p \text { iff } s \in v^-(p)$$;
3. $$\mathfrak {M}, s \Vdash ^+ \varphi \wedge \psi \text { iff } \mathfrak {M}, s \Vdash ^+ \varphi \text { and } \mathfrak {M}, s \Vdash ^+ \psi ,$$ $$ \mathfrak {M}, s \Vdash ^- \varphi \wedge \psi \text { iff } \mathfrak {M}, s \Vdash ^- \varphi \text { or } \mathfrak {M}, s \Vdash ^- \psi ;$$
4. $$\mathfrak {M}, s \Vdash ^+ \varphi \vee \psi \text { iff } \mathfrak {M}, s \Vdash ^+ \varphi \text { or } \mathfrak {M}, s \Vdash ^+ \psi ,$$ $$ \mathfrak {M}, s \Vdash ^- \varphi \vee \psi \text { iff } \mathfrak {M}, s \Vdash ^- \varphi \text { and } \mathfrak {M}, s \Vdash ^- \psi ;$$
5. $$\mathfrak {M}, s \Vdash ^+ \varphi \rightarrow \psi \text { iff } \forall y \in W, \text { s.th. } s \le y, \text { if } \mathfrak {M}, y \Vdash ^+ \varphi \text { then }\mathfrak {M}, y \Vdash ^+ \psi $$, $$\mathfrak {M}, s \Vdash ^- \varphi \rightarrow \psi \text { iff } \mathfrak {M}, s \Vdash ^+ \varphi \text { and } \mathfrak {M}, s \Vdash ^- \psi $$;
6. $$\mathfrak {M}, s \Vdash ^+ \lnot \varphi \text { iff } \mathfrak {M}, s \Vdash ^- \varphi $$, $$\mathfrak {M}, s \Vdash ^- \lnot \varphi \text { iff } \mathfrak {M}, s \Vdash ^+ \varphi $$.

#### Definition 4.13

($\textbf{NB}$ model) Let $\mathfrak {F}= \langle W, R \rangle$ be a frame, defined as above, but with $\le = \varnothing$. Let *R* be a reflexive and symmetric relation on *W*. Then, $\mathfrak {M} = \langle \mathfrak {F}, v\rangle$ is a $\textbf{NB}$-model iff $\mathfrak {F}$ is an $\textbf{NB}$-frame and $v:\texttt {Prop} \longrightarrow W$ is a valuation function.

#### Definition 4.14

(Truth in a ${\textbf {NB}}$ model) Let $\mathfrak {M} = \langle \mathfrak {F}, v\rangle$ be a ${\textbf {NB}}$-model for $\mathcal {L}_\bot$. Then, for every *s ∈ W*, we define $\mathfrak {M}, s \Vdash \varphi$ as supplementing the basic inductive clauses with the following:

- $$\mathfrak {M}, w \Vdash \varphi \rightarrow \psi \text { iff } \mathfrak {M}, w \nVdash \varphi \text { or }\mathfrak {M}, w \Vdash \psi $$;
- $$\mathfrak {M}, w \Vdash \lnot \varphi \text { iff there is a }w'\in W \text { such that }w'\in R(w) \text { and }\mathfrak {M}, w'\nVdash \varphi $$.

### Hyperintensionality Criteria and N-Logics

Before embarking in the endeavour of assessing the family of N-logics against the hyperintensionality criteria presented so far, we can observe some immediate relations between the criteria.

#### Observation 4.2

- In the presence of the deduction theorem, satisfaction of $\textbf{HYP}_L$ implies satisfaction of ${\textbf {HYP}}_{\texttt {int}}$ and $\textbf{HYP}_{\texttt {adm}}$ (but not viceversa).
- Satisfaction of $\textbf{HYP}_L$ and the presence of TC imply satisfaction of $\textbf{HYP}_{\textbf{CL}}$.
- Satisfaction of $\textbf{HYP}_{\textbf{CL}}$ implies satisfaction of $\textbf{HYP}_L$.

Next, I will state some results about satisfaction of the different criteria. As remarked above, ${\textbf {HYP}}_{\textbf{CL}}$ can be reduced as a special case of ${\textbf {HYP}}_L$ provided that the meaning of connectives can be restored in an adequate way.

#### Observation 4.3

(i) $${\textbf {N3}}$$ and $${\textbf {N4}}$$ satisfy $${\textbf {HYP}}_L$$.
(ii) ${\textbf {N3}}$ and ${\textbf {N4}}$ do not satisfy $\textbf{HYP}_\Box$.

#### Proof

Ad (i): from the fact that, in both ${\textbf {N3}}$ and ${\textbf {N4}}$, $\lnot (\varphi \rightarrow \psi ) \dashv \vdash \lnot (\lnot \varphi \vee \psi )$ but not $\lnot \lnot (\varphi \rightarrow \psi ) \dashv \vdash \lnot \lnot (\lnot \varphi \vee \psi )$. Since both ${\textbf {N3}}$ and ${\textbf {N4}}$ satisfy the deduction theorem, they both also satisfy ${\textbf {HYP}}_{\texttt {int}}$ and ${\textbf {HYP}}_{\texttt {adm}}$.

Ad (ii): both ${\textbf {N3}}$ and ${\textbf {N4}}$ are faithfully embeddable into $\text {S4}$, which is (strictly) non-hyperintensional. *□ *

#### Proposition 4.15

Both ${\textbf {N3}}$ and ${\textbf {N4}}$ satisfy ${\textbf {HYP}}_{CL}$.

#### Proof

First, define ${\textbf {N4}}^\bot$ and ${\textbf {N3}}^\bot$, by adding to the axioms of ${\textbf {N4}}$ and ${\textbf {N3}}$, formulated in $\mathcal {L}_\bot$, the following (Latkin, 2010):

$$\begin{aligned}&\bot \rightarrow \varphi \\&\varphi \rightarrow \lnot \bot \end{aligned}$$

Note that the intuitionistic negation can be defined in both ${\textbf {N4}}^\bot$ and ${\textbf {N3}}^\bot$ as $\sim \varphi : = \varphi \rightarrow \bot$ (Latkin, 2010). However, $\sim \varphi$ is not equivalent to $\lnot \varphi$ neither in ${\textbf {N4}}^\bot$ nor in ${\textbf {N3}}^\bot$. Then it can be seen that the *H* translation is a fixed and faithful translation between classical logic and both ${\textbf {N4}}^\bot$ and ${\textbf {N3}}^\bot$. Since ${\textbf {N4}}^\bot$ and ${\textbf {N3}}^\bot$ are not self-extensional, they will satisfy ${\textbf {HYP}}_{\textbf{CL}}$ with respect to the translation. *□ *

#### Proposition 4.16

${\textbf {NB}}$ satisfies $\textbf{HYP}_\Box '$, but not $\textbf{HYP}_\Box$.

#### Proof

As Avron and Zamansky (2020) show, ${\textbf {NB}}$ is faithfully embeddable in the modal logic ${\textbf {B}}$. However, ${\textbf {B}}$ does not satisfy Non-Hyp’, as noted also by Williamson (2006). Indeed, *\Box * creates a hyperintensional context in the sense of $\textbf{HYP}_\Box '$ in ${\textbf {B}}$. Note that

$$ \Box (p \equiv \top ) \supset \Box (\Box p \equiv \Box \top ) $$

is equivalent in ${\textbf {B}}$ to $\Box p \supset \Box \Box p$, which is not provable in ${\textbf {B}}$, since it fails in non-transitive frames. Hence, ${\textbf {B}}$ does not satisfy Non-Hyp’, and ${\textbf {NB}}$ satisfies $\textbf{HYP}_\Box '$.

On the other hand, assume ${\textbf {B}} \vdash \Box (\varphi \equiv \psi )$. Then, by the T-schema $\Box \varphi \supset \varphi$ and modus ponens ${\textbf {B}} \vdash \varphi \equiv \psi$. By self-extensionality, ${\textbf {B}} \vdash \theta (\varphi /p) \equiv \theta (\psi /p)$, and by necessitation ${\textbf {B}}\vdash \Box (\theta (\varphi /p) \equiv \theta (\psi /p))$. *□ *

#### Remark

Although ${\textbf {NB}}$ can be faithfully embedded into ${\textbf {B}}$, it corresponds to the local version of ${\textbf {B}}$, where necessitation is only an admissible rule, and not an inference rule. Indeed, we can define the ${\textbf {B}}$ necessity operator in ${\textbf {NB}}$ by means of negation and conditional, and the translation of the criterion $\textbf{HYP}_\Box$ is not satisfied because the logic is self-extensional.

Also, according to Williamson, the fact that ${\textbf {B}}$ is strictly necessity-hyperintensional boils down to the fact that “strict equivalence of two sentences at a point in a model no longer requires their intensions to coincide at that point”, because of the modal laws for ${\textbf {B}}$ (Williamson, 2006, p. 313). According to Williamson, this makes the phenomenon of necessity-hyperintensionality less interesting, because it is simply a consequence of the modal laws.

#### Proposition 4.17

(i) ${\textbf {NB}}$ does not satisfy $\textbf{HYP}_L$, and consequently it does not satisfy ${\textbf {HYP}}_{\textbf{CL}}$.
(ii) ${\textbf {NB}}$ does not satisfy ${\textbf {HYP}}_{\texttt {int}}$, but it trivially satisfies ${\textbf {HYP}}_{\texttt {adm}}$.

#### Proof

Ad (i): from the fact that ${\textbf {NB}}$ is self-extensional.

Ad (ii): for ${\textbf {HYP}}_{\texttt {int}}$, assume $\forall s: \mathfrak {M}, s \Vdash \varphi \leftrightarrow \psi$ and, seeking a contradiction, assume there is some *s'* and some *θ * such that $\mathfrak {M}, s'\nVdash \theta (\varphi /p) \leftrightarrow \theta (\psi /p)$. We prove the claim by induction on the complexity of *θ *. The only salient case is that in which *θ * is a negation. If $\mathfrak {M}, s'\nVdash \theta (\varphi /p) \leftrightarrow \theta (\psi /p)$, then, without loss of generality, $\exists s''. s'Rs''.\mathfrak {M},s'' \nVdash \varphi$ and $\forall s''.s'Rs'': \mathfrak {M}, s''\Vdash \psi$. This contradicts our assumption that $\forall s: \mathfrak {M}, s\Vdash \varphi$ iff $\mathfrak {M}, s \Vdash \psi$. For ${\textbf {HYP}}_{\texttt {adm}}$: since the conditional in ${\textbf {NB}}$ is extensional, satisfaction of ${\textbf {HYP}}_{\texttt {adm}}$ is simply equivalent to the claim that the ${\textbf {NB}}$ negation creates an intensional context. *□ *

#### Proposition 4.18

(i) ${\textbf {N}}, {\textbf {N}}^\circ , {\textbf {N}}^*, {\textbf {N}}^*_i$ satisfy ${\textbf {HYP}}_{\texttt {adm}}$, but do not satisfy ${\textbf {HYP}}_{\texttt {int}}$.
(ii) ${\textbf {N}}, {\textbf {N}}^\circ , {\textbf {N}}^*, {\textbf {N}}^*_i$ do not satisfy $\textbf{HYP}_L$, and consequently they do not satisfy ${\textbf {HYP}}_{\textbf{CL}}$.

#### Proof

Ad (i): following from the argument for ${\textbf {HYPE}}$ (${\textbf {N}}^*_i$) presented in Observation 3.2, and the fact that models of ${\textbf {HYPE}}$ are also models of ${\textbf {N}}, {\textbf {N}}^\circ , {\textbf {N}}^*$. For ${\textbf {HYP}}_{\texttt {int}}$: it is sufficient to reproduce an argument similar to that of Observation 3.1.

Ad (ii): Follows from the fact that all Došen-style logics are self-extensional [which in turn follows from them having contraposition as a rule, see Avron and Zamansky (2020, Proposition 3.2)]. *□ *

#### Proposition 4.19

${\textbf {N}}, {\textbf {N}}^\circ , {\textbf {N}}^*, {\textbf {N}}^*_i$ do not satisfy $\textbf{HYP}_\Box$.

#### Proof

As noted by Odintsov and Wansing (2021), ${\textbf {HYPE}}$ can be faithfully embedded into a normal bi-modal logic $\text {S4*KD!B}$ with a conditional corresponding to a $\text {S4}$ modality.

The relevant semantic clauses for the logic $\text {S4*KD!B}$ are the following:

$$\begin{aligned} \mathfrak {M}, w \Vdash \lnot _c \varphi&\text { iff } \mathfrak {M}, w \nVdash \varphi \\ \mathfrak {M}, w \Vdash \Box \varphi&\text { iff } \forall w'. w \le w' : \mathfrak {M}, w' \Vdash \varphi \\ \mathfrak {M}, w \Vdash \blacksquare \varphi&\text { iff } \forall w'(wRw'): \mathfrak {M}, w' \Vdash \varphi \end{aligned}$$

The other Došen-style logics can be embedded into similar modal logics, just changing the frame conditions for negation.

There is a translation $\tau :\mathcal {L} \longrightarrow \mathcal {L}^{\Box , \blacksquare }$ as follows:

$$\begin{aligned} \tau (\top )&= \top , \quad \tau (\bot ) = \bot \\ \tau (p)&= \Box p, \text { for all } p \in \texttt {Prop}\\ \tau (\varphi \# \psi )&= \tau (\varphi ) \# \tau (\psi ), \# \in \{\wedge , \vee \}\\ \tau (\varphi \rightarrow \psi )&= \Box (\tau (\varphi ) \supset \tau (\psi ))\\ \tau (\lnot \varphi )&= \blacksquare \lnot _c \tau \varphi . \end{aligned}$$

We refer to the consequence relation for $\text {S4*KD!B}$ as $\vdash _\Box$ Then we have, for all ${\textbf {L}} \in \{{\textbf {N}}, {\textbf {N}}^\circ , {\textbf {N}}^*, {\textbf {N}}^*_i\}$:

$$\begin{aligned}&\vdash _{{\textbf {L}}} (\varphi \leftrightarrow \psi ) \rightarrow (\theta (\varphi /p) \leftrightarrow \theta (\psi /p) \text { since the logics are self-extensional}\\&\vdash _{\Box } \Box [(\varphi \equiv \psi )^\tau \supset ((\theta (\varphi /p) \equiv \theta (\psi /p))^\tau ]\\&\vdash _\Box \Box (\varphi \equiv \psi )^\tau \supset \Box ((\theta (\varphi /p) \equiv \theta (\psi /p))^\tau \text { by the S4 law} \end{aligned}$$

The last line entails that $\text {S4*KD!B}$ and the related logics are strictly non-hyperintensional, hence also non-hyperintensional. *□ *

The above results can be conveniently summarised in the following table.

**Table:** 

-	${HYP}_{{L}}$	${HYP}_{{CL}}$	${HYP}_\Box '$	${HYP}_{\texttt{adm}}$
**N**	✗	✗	✗	✓
$N^{\circ }$	✗	✗	✗	✓
$N^*$	✗	✗	✗	✓
$N^*_i$	✗	✗	✗	✓
**NB**	✗	✗	✓	n/a
**N4**	✓	✓	✗	✓
**N3**	✓	✓	✗	✓

## Hyperintensionality and Contraposition

In the previous section, we have applied the criteria for hyperintensionality defined in this paper against a family of logics with different behaviours for the negation and conditional, noting that these satisfy different criteria for hyperintensionality. I will here hint at a general interpretation of the results presented so far.

According to Avron and Zamansky (2020, proposition 3.2), all logics extending the negation-free fragment of intuitionistic or classical logic which admit contraposition as a rule are self-extensional, hence not Odintsov-Wansing hyperintensional. However, as we saw with the case of ${\textbf {M}}^{{\textbf {f}}}$, the failure of self-extensionality, and hence the satisfaction of Odintsov-Wansing hyperintensionality, is not only due to the absence of the contraposition rule in a logic.

On the other hand, we seem to be able to attribute the satisfaction of admissible hyperintensionality to failures of contraposition at the level of the semantics. In particular, all the logics which we are considering satisfying admissible hyperintensionality have contraposition as an admissible rule, but not as a derivable rule.^24^ Hence, the contraposition axiom is not licensed. This can be explained by the interaction between the intuitionistic conditional and negation understood as a modal operator. For instance, in ${\textbf {NB}}$, where the conditional is fully extensional, even though the negation acts as a modal operator we do not observe this behaviour (because ${\textbf {HYP}}_{\texttt {adm}}$ collapses to intensionality).

What makes a system of pure logic hyperintensional, then, does not seem to be non-self-extensionality per se, but the presence of “asymmetric” operators or connectives. Then, either a negation which does not respect contraposition, to a certain degree—so either contraposition fails at the level of the proof system, making the logic non-self-extensional, or at the level of the semantics—or a modality creating an asymmetrical context, as we saw in the different cases of ${\textbf {M}}^{{\textbf {f}}}$ and ${\textbf {NB}}$. Indeed, as Speranski (2022, Theorem 3.5) argues, the addition of contraposition as an axiom to the logic ${\textbf {N}}^*$ makes it collapse to classical logic. Similar results are obtained for other Došen style logics with respect to minimal logic and intuitionistic logic.

There does not seem, then, to be a natural stopping point in the continuum between intensionality and interpreted syntax. Hyperintensionality can be tentatively diagnosed as a degree of asymmetry exceeding the resources of intensionality. In a sense, this brings us back to square one: we are simply defining hyperintensionality as something “more than intensional”. Hyperintensionality appears whenever the specification of intension which we can have in a given logic is not enough to warrant intersubstitutivity salva veritate, or, simply, when the condition that is meant to express co-intensionality fails to do so properly.^25^ However, since there might be several ways to generalise the notion of intension when moving to logics with state-based semantics, there is no one way to specify what counts as a hyperintension. Hyperintensionality does coincide with a mismatch between sameness of semantic content for two expressions and some equivalence meter which is meant to represent sameness of intension. But that meter does not necessarily have to be interderivability.

This analysis took a small step in the direction of more accurately characterising hyperintensionality for logics. An immediate next step would be to generalise this analysis both to formal theories (e.g. theories of truth and properties) and to logics with a non-Tarskian consequence relation. These are two natural generalisations. On the one hand, as anticipated in the introduction, property identity is traditionally taken to generate a hyperintensional context. As the remarks about Nicolai and Stern’s modal logics seem to suggest, there might be a sense in which a truth operator generates a hyperintensional context. Similarly, the analysis can be extended to some phenomena in set or class theory, such as, for example, forcing names, as argued for by Barton (2024). On the other hand, when considering substructural logics with non-Tarskian consequence relations, it is a natural question to ask whether their meta-inferential consequence relations have elements of hyperintensionality.

## Footnotes

1 Although Nolan (2014) presents a different view, understanding hyperintensionality as a feature of non-representational phenomena as well as representational ones; see also Lenta (2023).

2 Axiomatic theories can be said to be hyperintensional too, when they have a predicate, relation or operator creating a hyperintensional context, understood as a context in which *terms* with the same intension cannot be intersubstituted salva veritate. For example, formal theories of properties canonically display hyperintensional features, since property identity is normally considered to be hyperintensional (see Bealer, 1982; Dorr, 2016). More recently, Barton has suggested that even classical set theory (normally considered to be the extensional theory *par excellence*) displays, when considering forcing names, a radical kind of indeterminate naming which may be understood hyperintensionally (Barton, 2024).

3 In the following I will use the convention of indicating which logic the consequence relation refers to with a subscript. When the consequence relation under consideration is clear, the subscript will be omitted.

4 See the bilateral treatment of ${\textbf {N4}}$ in Drobyshevich (2021).

5 Stern’s claims are directed to the hyperintensionality of Nelson’s partial logic ${\textbf {N3}}$, as presented by Thomason (1969), which is also hyperintensional in Odintsov and Wansing’s sense, and, like ${\textbf {N4}}$, satisfies the replacement property.

6 As noted by Korbmacher (2023, p. 14), also the logic of exact entailment, which is sound and complete with respect to (inclusive) exact truthmaker semantics, is hyperintensional in the $\textbf{HYP}_L$ sense, because negation is not congruential. Indeed, exact equivalence (i.e., mutual exact entailment) corresponds to sameness of truthmaker content, and not sameness of semantic content, understood as equivalence of truthmakers and falsemakers.

7 Note, further, that every non-classical logic which counts as hyperintensional on the criterion $\textbf{HYP}_L$ will be hyperintensional with respect to classical logic in this naïve sense, while still falling prey to the objection from meaning of connectives.

8 Korbmacher (2023, footnote 19) applies the definition of hyperintensionality with respect to classical logic in the “naïve” sense as well when considering the logic of exact entailment (see Fine & Jago, 2019).

9 Another, simpler, example of this phenomenon, which works for all partial modal logics considered by Nicolai and Stern, is $\top \dashv \vdash p \vee \lnot p$, which are not intersubstitutable inside the necessity operator. I would like to thank an anonymous referee for bringing this to my attention.

10 Indeed, Leitgeb (2007) stipulates as one of the desiderata for a truth theory that the inner and outer logic should coincide. However, this is not the case for many theories of truth, such as ${\textbf {KF}}$-style theories or most supervaluational theories. While there are theories of truth which have been dubbed as hyperintensional, such as Stern’s Strong Kleene supervaluations over ${\textbf {N3}}$ (Stern, 2025), this hyperintensional behaviour comes from the logic itself being non-self-extensional, and not from the truth predicate licensing non-congruentiality.

11 Obtained by adding to inclusive semantics an assumption of non-vacuity (every statement has at least one truthmaker and one falsemaker) and “convexity” (if *p*, *q* are exact truthmakers/falsemakers and $p \sqsubseteq r \sqsubseteq q$, where *⊑ * indicates parthood, then *r* is an exact truthmaker/falsemaker) (Fine, 2016).

12 For *→ * to be a conditional connective for ${\textbf {L}}$, it must satisfy modus ponens.

13 Although, technically speaking, the conditional in connection with the modal operator in ${\textbf {M}}^{{\textbf {f}}}$ expresses something stronger than necessary equivalence. Also, the other truth logics discussed by Nicolai and Stern would turn out as hyperintensional despite the fact that necessary equivalents can be substituted salva veritate in every context. I would like to thank an anonymous referee for raising these points.

14 That is, contexts indexed by a hyperintensional operator, in this case “seeing”.

15 I will be neutral with respect to a choice of metaphysical constraints and use the word “state” instead of “situation” or “possibility”.

16 This criterion can also be expressed semantically, establishing an equivalence between so-called classical models of ${\textbf {HYPE}}$ and models of classical logic, and between classical logic and the conditional-free consequence relation for so-called “worldly states” of ${\textbf {HYPE}}$ models, i.e. states behaving like classical possible worlds, without gaps or gluts (Leitgeb, 2019, p. 378).

17 In fact, recapture results for ${\textbf {HYPE}}$ are also available in the sequent calculus $\mathbf {G1h_{cd}}$, as shown by Fischer et al. (2023, Lemma 1).

18 I would like to thank an anonymous referee for raising this point.

19 Furthermore, recapture results may pose a problem if we wanted to assess the hyperintensionality of first-order theories [see Nicolai (2022), see also discussion in Fiore and Rosenblatt (2023)].

20 See (Fine, 2016, p. 210).

21 For logics whose conditional is evaluated locally, this criterion “collapses” to simply a criterion for intensionality.

22 Following from the fact that $\varphi \rightarrow \psi \nvdash _{{\textbf {H}}} \lnot \psi \rightarrow \lnot \varphi$.

23 See also Odintsov and Wansing (2021) for a presentation of Vakarelov’s framework.

24 See also Odintsov (2010) for remarks on this about Došen-style logics.

25 I would like to thank an anonymous referee for raising this point.
